# Good prediction of treatment responses to neoadjuvant chemoradiotherapy for esophageal cancer based on preoperative inflammatory status and tumor glucose metabolism

**DOI:** 10.1038/s41598-021-90753-y

**Published:** 2021-06-02

**Authors:** Chuan Li, Jing-Wei Lin, Hui-Ling Yeh, Cheng-Yen Chuang, Chien-Chih Chen

**Affiliations:** 1 Department of Radiation Oncology, Taichung Veterans General Hospital, No.1650, Sect. 4, Taiwan Boulevard, Taichung, 407 Taiwan; 2 Ph.D. Program in Translational Medicine, National Chung Hsing University, Taichung, Taiwan; 3 Division of Thoracic Surgery, Department of Surgery, Taichung Veterans General Hospital, Taichung, Taiwan; 4 Department of Radiation Oncology, Chiayi Branch, Taichung Veterans General Hospital, Chiayi City, Taiwan

**Keywords:** Cancer, Oncology

## Abstract

To develop a tool for predicting pathologic complete response (pCR) after neoadjuvant chemoradiotherapy (neoCRT) in patients with esophageal cancer by combining inflammatory status and tumor glucose metabolic activity. This study included 127 patients with locally advanced esophageal cancer who had received neoCRT followed by esophagectomy from 2007 to 2016. We collected their neutrophil–lymphocyte ratio (NLR) and standardized uptake value (SUV) obtained from fluorodeoxyglucose positron emission tomography (PET/CT) before and after neoCRT. Univariate and multivariate logistic regression analyses were performed to identify potential predictive factors for pCR. Sensitivity, specificity, positive predictive value (PPV), and negative predictive value (NPV) of predictors were calculated. Between pCR and non-pCR groups, there were no statistically significant differences in patient characteristics, such as sex, age, site, and clinical T/N stage. Multivariate analyses identified four independent predictors for pCR, including pre-OP NLR < 5.4 [OR 11.179; 95% CI 8.385–13.495; p = 0.003], NLR change (ΔNLR) < 3 [OR 4.891; 95% CI 2.274–9.180; p = 0.005], changes in SUV (ΔSUV) > 7.2 [OR 3.033; 95% CI 1.354–6.791; p = 0.007], and SUV changes ratio (ΔSUV ratio) > 58% [OR 3.585; 95% CI 1.576–8.152; p = 0.002]. ΔNLR had the highest accuracy and NPV (84.3% and 90.3%, respectively). Combined factors of ΔNLR < 3 and ΔSUV ratio > 58% had the best PPV for pCR (84.8%). Inflammatory status (ΔNLR) and tumor glucose metabolic activity (ΔSUV ratio), when considered together, constitute a promising low-invasive tool with high efficacy for prediction of treatment response before surgery.

## Introduction

Esophageal cancer was the 7th most common cancer worldwide in 2018 and was ranked the 6th most common cause of cancer-related mortality^1^. The 5-year overall survival rate (OS) of patients with esophageal cancer treated with surgery alone is ≤ 25%. More than 20% of patients have microscopic positive resection margin after primary surgery, and 31% of patients have locoregional failure^2^. In locally advanced esophageal cancer, neoadjuvant chemoradiotherapy (neoCRT) followed by surgery improves survival and local control^3–6^. The pathological complete response rate (pCR) of this approach ranges from 13 to 47%^7,8^. Esophagectomy is a major surgery, which results in morbidity, a mortality rate of up to 5%, and impaired quality of life^9^. In older patients with multiple comorbidities, achieving pCR, the benefit of esophagectomy could be less than the risk of complications. A more precise preoperative prediction of treatment response may help decision-making in the organ-preserving approach, thereby potentially reducing postoperative morbidity and mortality.

Changes associated with inflammation are crucial in tumor development and progression^10^. The neutrophil-to-lymphocyte ratio (NLR) is an inflammatory-based score with prognostic values in some cancers, such as esophageal cancer, cervical cancer, and hepatocellular cancer^11–13^. Elevated NLR is associated with poor survival outcomes in patients with esophageal cancer treated with neoCRT followed by surgery^11^. However, it remains unclear whether NLR can predict tumor response after neoCRT.

Apart from inflammatory factors, functional images, such as Fluorodeoxyglucose Positron Emission Tomography (FDG-PET/CT), are also indicators of responses to neoCRT. FDG-PET/CT uses the radiolabeled glucose analog 18F-fluorodeoxyglucose (FDG) to detect the glucose metabolic activity of cancer cells. Changes in FDG uptake after neoCRT reflect changes in glucose metabolic activity by tumor cells which can be used to predict treatment responses^14,15^. A systemic review demonstrated the ratio of change in maximum standardized uptake value at one hour (ΔSUV ratio) after neoCRT is associated with treatment response. However, the cut-off point of the ΔSUV ratio varies, and the sensitivity and specificity of the ΔSUV ratio are heterogeneous^14^. It is risky to predict treatment response after neoCRT based solely on tumor glucose metabolic activity.

Here we aimed to determine the predictive values of combined inflammatory status and tumor glucose metabolism in patients with esophageal cancer treated with neoCRT followed by surgery.

## Materials and methods

### Study population

We first reviewed patients with histologically proven squamous cell carcinoma (SCC) of the esophagus at clinical stage T2-4N0-3M0 based on the 8th edition of the American Joint Committee on Cancer staging manual. Patients who received neoCRT followed by surgery were then included for analysis. The exclusion criteria were: (a) histology other than SCC; (b) distant metastasis at initial diagnosis; (c) synchronous malignancy; (d) previous history of malignancies within five years, except non-melanoma skin cancer or adequately treated carcinoma in situ of the cervix; (e) concurrent chemotherapy other than cisplatin and 5-fluorouracil (5-FU) and (f) incomplete treatment.

Our study was approved by the Institutional Review Board of Taichung Veterans General Hospital. Written informed consent was obtained from all participating patients. All patients were treated according to the National Comprehensive Cancer Network (NCCN) guideline for esophageal cancer.

### Treatment protocol

Pretreatment workup included physical examination, hematologic and biochemistry profile, endoscopic esophageal tumor biopsy, chest computed tomography scan (CT), FDG-PET/CT, transesophageal endoscopic ultrasound, and bronchoscopy.

Radiotherapy was given with a total dose from 50 to 50.4 Gy in 1.8 to 2.0 Gy per fraction, five daily fractions per week.

During the treatment period of radiotherapy, concurrent chemotherapy was given intravenously with cisplatin at 20 mg/m^2^ drip for 2 h and fluorouracil 800 mg/m^2^ for 24 h on Days 1–4 (cycle 1), and Days 29–32 (cycle 2).

A second PET-CT scan was performed within 3–4 weeks after the completion of neoCRT to evaluate the treatment response before surgery. Patients received surgery within 4–6 weeks after completion of neoCRT. The surgery procedure included esophagectomy, lymph node dissection, and esophagus reconstruction with the gastric tube. Pathologic reports included differentiation, margin status, tumor regression grade (TRG), and nodal metastasis. Pathological complete response (pCR) was defined as the absence of tumor cells in the primary tumor and all dissected lymph nodes.

### Inflammatory status and tumor glucose metabolic activity

Data on inflammatory status were extracted from medical records. The neutrophil-to-lymphocyte ratio (NLR) was defined as the absolute neutrophil count divided by the absolute lymphocyte count. All differential counts were taken within 1 week before neoCRT (Pre-CRT NLR) and 1 week before surgery (Pre-OP NLR). The change of NLR (ΔNLR) was defined as pre-OP NLR substrate pre-CRT NLR. Tumor glucose metabolic activity was defined as the maximum standardized uptake value at one hour of primary esophageal tumor which was obtained from PET/CT before neoCRT (pre-CRT SUV) and after neoCRT (pre-OP SUV). The change of SUV (ΔSUV) after neoCRT was defined as pre-CRT SUV minus pre-OP SUV. The ratio of change in SUV (ΔSUV ratio) was defined as ΔSUV divided by pre-CRT SUV.

### Statistical analyses

Patients’ characteristics’ included sex, age, histological differentiation, site, clinical T/N classification, pre-treatment tumor length, time interval between neoCRT to surgery, and numbers of dissected lymph nodes. Between pCR and non-pCR groups, the chi-square test or Fisher's exact test was used to compare categorical variables, and the 2-sample t-test was used to compare continuous variables. The associations between clinical parameters and pCR were evaluated by univariate and multivariate analyses using the logistic regression model. Variates with p < 0.05 and confounders, including clinical T stage and clinical N stage in the univariate analysis, were further explored using multivariate analysis. Receiver operating characteristic (ROC) curves for pCR prediction were used to verify the best cut-off point for pre-CRT NLR, pre-OP NLR, ΔNLR, pre-CRT SUV, pre-OP SUV, ΔSUV, and SUV ratio. Sensitivity, specificity, positive predictive value (PPV), negative predictive value (NPV), and accuracy were calculated for both tumor glucose metabolic and inflammatory factors.

Survival outcomes were analyzed using the Kaplan–Meier method and log-rank tests. OS was measured from the date of biopsy until death from any cause or time of the most recent follow-up. Progression-free survival (PFS) started from the date of biopsy until death or disease progression. Data were analyzed statistically using SPSS software (version 25.0, IBM, NY).

### Ethical approval

This study was approved by the Institutional Review Board of Taichung Veterans General Hospital. (IRB number: CE19242A).

### Informed consent

Written informed consent was obtained from all participating patients. All patients were treated according to the National Comprehensive Cancer Network (NCCN) guideline for esophageal cancer.

## Results

We retrospectively analyzed 127 patients who had received neoCRT between October 2007 and July 2016. All of them completed neoCRT and then received surgery. Patients’ characteristics are shown in Table 1. The mean age of the entire cohort was 54.77 ± 8.22 years (mean ± SD). The time interval between the date of completing neoCRT and the date of the surgery was 34.71 ± 7.78 days (mean ± SD). The median time interval between the date of completing neoCRT and the date of the second PET-CT was 22 days (IQR 19–25 days). The median follow-up duration of the surviving patients was 59.7 months. The 5-year OS of the entire cohort was 59.1%. Of the 127 patients, 57 of them showed pathological complete responses after neoCRT with a pCR rate of 44.9%.

**Table 1:** Patients’ characteristics.

		Total N = 127	pCR N = 57	Non-pCR N = 70	p Value
Sex		F:5/M:122	F:3/M:54	F:2/M:68	0.656
Age (years)	Range	35–75	35–75	35–71	0.983
	Mean ± SD	54.77 ± 8.22	54.75 ± 9.10	54.85 ± 7.45	
Site	Cervical	1	0	1	0.433
	**Thoracic**				
	U	15	8	7	
	M	24	13	11	
	L	80	34	46	
	2 sites	7	2	5	
cT	1	1	0	1	0.152
	2	3	3	0	
	3	120	52	68	
	4	3	2	1	
cN	0	6	4	2	0.765
	1	70	31	39	
	2	46	20	26	
	3	5	2	3	
Primary tumor	Range	2–20	2–19	2–20	0.445
Length (cm)	Mean ± SD	6.85 ± 3.13	6.61 ± 2.68	7.01 ± 3.45	
Time to surgery	Range	15–65	18–63	15–65	0.766
(day)	Mean ± SD	34.71 ± 7.78	33.80 ± 7.72	33.28 ± 7.86	
Histology	Others	59	28	31	0.930
	PD SCC	68	29	39	
Dissected LN (N)	Range	5–93	7–9	5–74	0.878
	Mean ± SD	31.95 ± 15.24	32.18 ± 17.77	31.83 ± 12.89	

*pCR* pathological complete response, *N* number, *F* female, *M* male, *SD* standard deviation, *U* upper thoracic, *M* middle thoracic, *L* lower thoracic, *cT* clinical T classification, *cN* clinical N classification, *PD SCC* poor differentiated squamous cell carcinoma, *LN* lymph nodes.

Clinical features, which were similar between pCR and non-pCR groups, included sex, age, site, clinical T/N stage, histology, time duration to surgery, and numbers of dissected LN.

**Figure 1:**
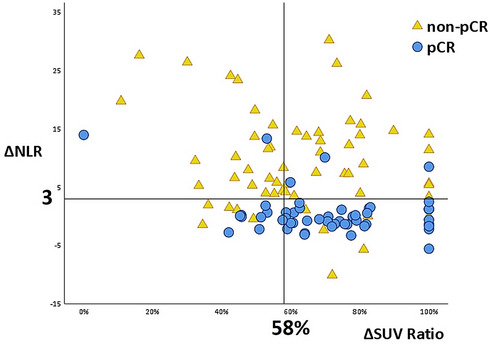
Distribution of ΔNLR and ΔSUV ratio in patients with or without pCR. *ΔNLR* the change of neutrophil-to-lymphocyte ratio, *pCR* pathological complete response, *OS* overall survival, *PFS* progression-free survival, *ΔSUV ratio* the change in maximum standardized uptake value at 1 h.

The potential predictors of pCR estimated by univariate and multivariate analyses are shown in Table 2. Multivariate analyses identified four independent predictors for pCR, including pre-OP NLR < 5.4 [OR 11.179; 95% CI 8.385–13.495; p = 0.003], NLR change (ΔNLR) < 3 [OR 4.891; 95% CI 2.274–9.180; p = 0.005], changes in SUV (ΔSUV) > 7.2 [OR 3.033; 95% CI 1.354–6.791; p = 0.007], and SUV changes ratio (ΔSUV ratio) > 58% [OR 3.585; 95% CI 1.576–8.152; p = 0.002]. ROC curves for pre-OP NLR, ΔNLR, ΔSUV, and ΔSUV ratio in predicting pCR were plotted in Supplementary Information 1. The optimized cut-off values for pre-OP NLR and ΔNLR were 5.4 and 3, respectively. The best cut-off points for ΔSUV and ΔSUV ratio were 7.2 and 58%, respectively. The prediction efficacy of the inflammatory (pre-OP NLR and ΔNLR) and the tumor glucose metabolic factors (ΔSUV and ΔSUV ratio) are shown in Table 3 and 4. The distribution of ΔNLR and ΔSUV ratio in patients with or without pCR is shown in Fig. 1. ΔNLR showed the highest accuracy and NPV (84.3% and 90.3%, respectively). In tumor glucose metabolic markers, ΔSUV ratio showed great sensitivity and NPV (77.8% and 72.7%, respectively). Combined factors of inflammatory status (ΔNLR < 3) and tumor glucose metabolic marker (ΔSUV ratio > 58%) gave the best PPV for pCR (84.8%). Higher PPV had a lower risk of false positive for the prediction of pCR. The risk of pathological residual tumor was 21.5% if using only ΔNLR < 3. This risk could be reduced to 15.2% with the combination of ΔNLR < 3 and ΔSUV ratio > 58%. The odds ratio of combined condition of ΔNLR < 3 and ΔSUV ratio > 58% was 21.543 (95% CI 8.047–57.670). High ΔNLR and low ΔSUV ratio were associated with residual tumor after neoCRT [OR 11.333, 95% CI 3.196–40.184].

**Table 2:** Results of univariate and multivariate analyses based on logistic regression on pCR.

	Univariate		Multivariate	
	OR (95% CI)	p value	OR (95% CI)	p Value
Sex (Ref: M)	1.889 (0.305–11.711)	0.494		
Age	1.000 (0.958–1.043)	0.983		
Histology (Ref: MD)	0.967 (0.454–2.058)	0.930		
Location (Ref: U/M)	0.638 (0.245–1.361)	0.245		
**Clinical T stage**				
cT1, cT2 (Ref)	1	0.250	1	0.249
cT3, cT4	0.261 (0.026–2.579)		0.219 (0.017–2.897)	
**Clinical N stage**				
cN0, cN1 (Ref)	1	0.746	1	0.821
cN2, cN3	0.889 (0.435–1.8160)		0.904 (0.375–2.178)	
Primary tumor length	0.956 (0.852–1.073)	0.444		
Time of CCRT to OP	1.016 (0.985–1.048)	0.322		
Pre-CCRT NLR (as continuous variable)	1.030 (0.860–1.235)	0.746		
Pre-OP NLR	0.816 (0.750–0.887)	< 0.001		
**Pre-OP NLR**				
≧5.4 (Ref)	1	< 0.001	1	0.003
< 5.4	11.333 (8.486–13.632)		11.179 (8.385–13.495)	
ΔNLR (as continuous variable)	0.833 (0.771–0.900)	< 0.001		
**ΔNLR**				
≧ 3 (Ref)	1	< 0.001	1	0.005
< 3	4.010 (2.152–9.128)		4.891 (2.274–9.180)	
Pre-CCRT SUV	1.053 (0.973–1.139)	0.198		
Pre-OP SUV	0.859 (0.724–1.020)	0.083		
ΔSUV (as continuous variable)	1.073 (0.997–1.154)	0.059		
**ΔSUV > 7.2**				
≦ 7.2 (Ref)	1	0.009	1	0.007
> 7.2	2.876 (1.303–6.344)		3.033 (1.354–6.791)	
ΔSUV ratio (as continuous variable)	1.021 (1.003–1.039)	0.025		
**ΔSUV ratio > 58%**				
≦ 58% (Ref)	1	0.003	1	0.002
> 58%	3.394 (1.517–7.591)		3.585 (1.576–8.152)	

*OR* odds ratio, *95% CI* 95% confidence interval, *M* male, *MD* moderately differentiated, *U* upper thoracic, *M* middle thoracic, *cT* clinical T classification, *cN* clinical N classification, *CCRT* concurrent chemoradiotherapy, *OP* operation, *pre-CCRT NLR* neutrophil–lymphocyte ratio before concurrent chemoradiotherapy, *pre-OP NLR* neutrophil–lymphocyte ratio before operation, *ΔNLR* the change of neutrophil–lymphocyte ratio before and after concurrent chemoradiotherapy, *Hb* hemoglobulin, *Alb* albumin, *LDH* lactate dehydrogenase, *CRP* C-reactive protein, *pre-CCRT SUV* maximum standardized uptake value of the esophageal tumor before concurrent chemoradiotherapy, *pre-OP SUV* maximum standardized uptake value of the esophageal tumor before operation, *ΔSUV* the change of maximum standardized uptake value of the esophageal tumor before and after concurrent chemoradiotherapy.

**Table 3:** Predictive efficacy of inflammatory factors and tumor glucose metabolism for pCR.

	Sensitivity (%)	Specificity (%)	PPV (%)	NPV (%)	Accuracy (%)
Pre-OP NLR < 5.4	84.2	80.2	77.4	87.7	82.7
ΔNLR < 3	89.5	80.0	78.5	90.3	84.3
ΔSUV > 7.2	75.9	56.4	56.0	70.4	60.5
ΔSUV ratio > 58%	77.8	49.2	56.0	72.7	62.2

*PPV* positive predict value, *NPV* negative predict value, *pre-OP NLR* neutrophil–lymphocyte ratio before operation, *CCRT* concurrent chemoradiotherapy, *OP* operation, *pre-CCRT NLR* neutrophil–lymphocyte ratio before concurrent chemoradiotherapy, *pre-OP NLR* neutrophil–lymphocyte ratio before operation, *ΔNLR* the change of neutrophil–lymphocyte ratio before and after concurrent chemoradiotherapy, *ΔSUV* the change of maximum standardized uptake value of the esophageal tumor before and after concurrent chemoradiotherapy.

**Table 4.** Predictive value of combined ΔNLR and ΔSUV for pCR.

	pCRpCR	Non-pCR
ΔNLR < 3 & ΔSUV ratio > 58% (N = 46)	39 (84.8%)	
ΔNLR > 3 & ΔSUV ratio < 58% (N = 29)		26 (89.7%)

*pCR* pathological complete response, *ΔNLR* the change of neutrophil–lymphocyte ratio before and after concurrent chemoradiotherapy, *ΔSUV ratio* the change of maximum standardized uptake value of the esophageal tumor before and after concurrent chemoradiotherapy to pre-chemotherapy.

**Figure 2:**
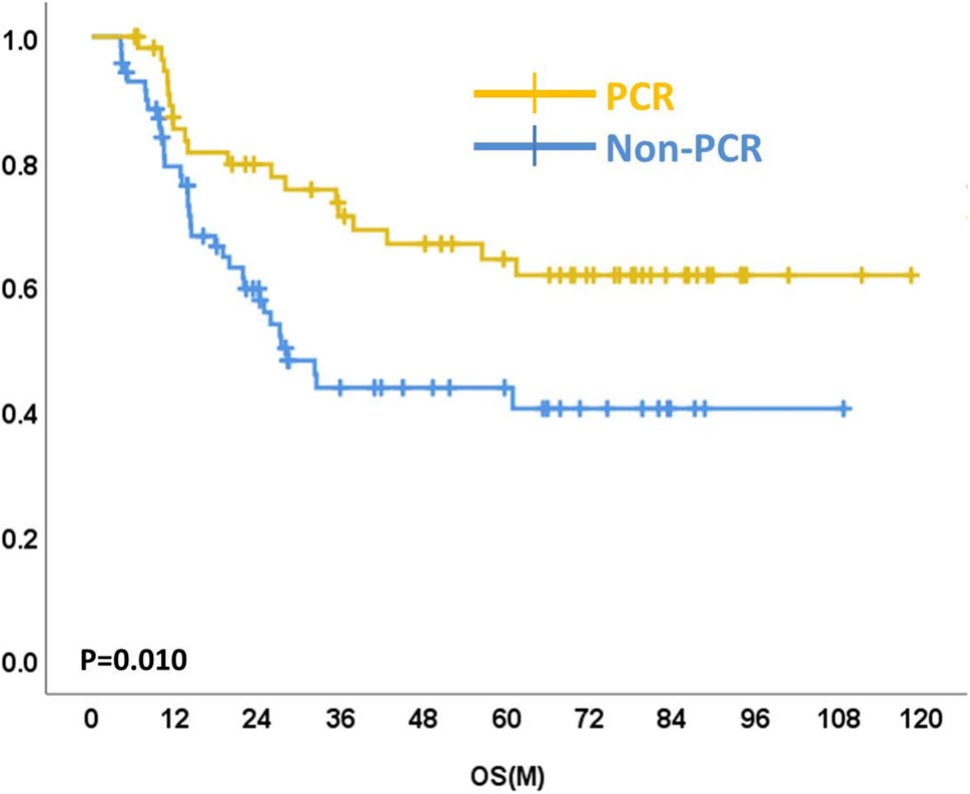
Overall survival curves of esophageal cancer patients with pCR and non-pCR.

**Figure 3:**
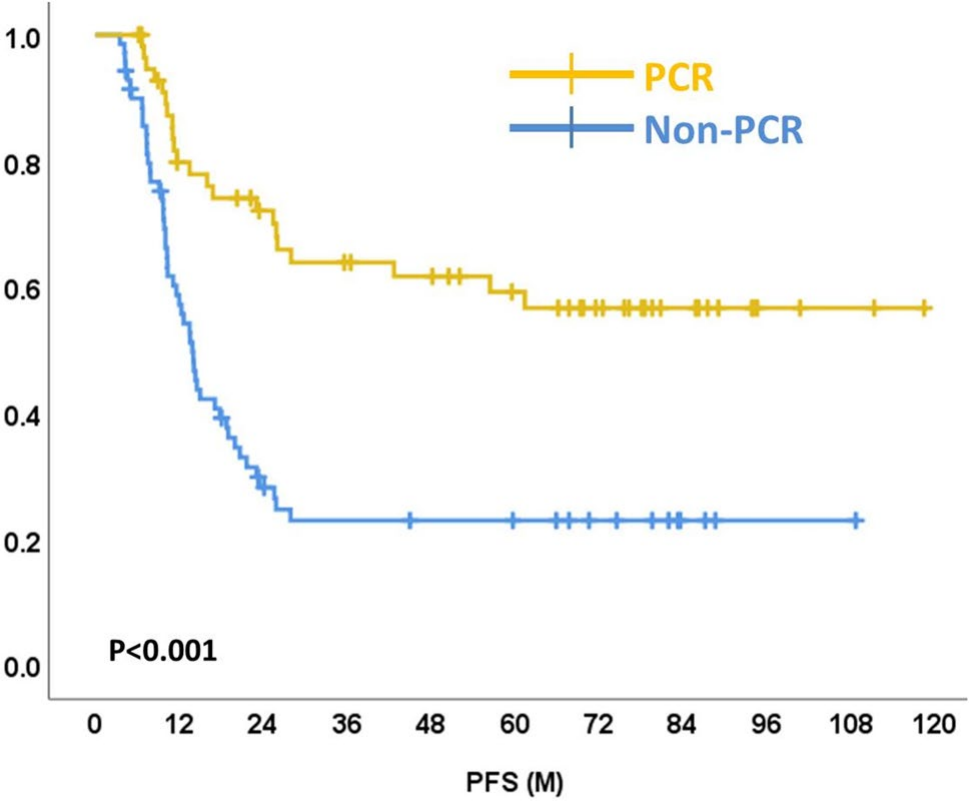
Progression-free survival curves of esophageal cancer patients with pCR and non-pCR.

Survival curves of pCR and non-pCR are shown in Figs. 2 and 3. The 5-year OS in the pCR group was 68.4% compared with 51.4% in the non-pCR group (p = 0.010). The 5-year PFS in the pCR group was 63.6% compared with 27.1% in the non-pCR group (p < 0.001).

## Discussion

We reviewed the data of patients with locally advanced esophageal SCC to determine the predictive value of combining the conditions of inflammatory status and tumor glucose metabolism. We found that patients with both low ΔNLR (< 3) and high ΔSUV ratio (> 58%) were associated with a higher probability of pCR [OR 21.543, (95% CI 8.047–57.670)]. The PPV was 84.8% based on the combined condition of a low ΔNLR and a high ΔSUV ratio. Patients with high ΔNLR and low ΔSUV showed a higher probability of having residual tumors. To the best of our knowledge, this is the first study to assess the accuracy of combining both inflammatory status and tumor glucose metabolic activity for predicting pCR in patients with esophageal SCC after neoCRT. NLR was calculated with absolute neutrophil and lymphocyte counts derived from differential counts, which is a low-cost and low-invasive method. SUV obtained from PET-CT is also a minimally invasive procedure. After neoCRT, ΔNLR combined with ΔSUV can help physicians predict the treatment response before surgery.

In previous studies, high NLR was found to be associated with poor survival outcomes in esophageal cancer patients^11^. However, the time points of NLR vary among different reports. Sharaiha et al. analyzed esophageal cancer patients who received surgery with or without neoCRT. They found that high pre-OP NLR (> 5) was associated with poor OS and PFS^16^. Feng et al. also confirmed this result with a different cut-off point of NLR (> 3.5)^17^. In a few studies, high pre-CRT NLR was reported to have a negative impact on survival in patients treated with neoCRT followed by surgery^18^. Yoo et al. reported that a high pre-CRT NLR was also a negative predictor in the definitive CCRT setting^19^.

Some recent studies have used NLR to predict pCR after neoadjuvant treatments. McLaren et al. analyzed 60 esophageal cancer patients and found that a high pre-CRT NLR was a negative predictor of pCR^20^. Hyder et al. reported the smaller the increase in NLR (< 4.6), the higher the probability of achieving pCR^21^. In these studies, an association was found between NLR and pCR. However, these studies involved only a few cases and their predictive efficacy is unclear. In our analysis, we reviewed 127 patients and found that lower NLR increase (ΔNLR < 3) had a 4- to fivefold probability of achieving pCR [OR 1.891; 95% CI 2.274–9.180; p = 0.005].

In the published reports on esophageal cancer patients, changes in pre-OP PET-CT parameters were found to reflect the tumor response after neoCRT and have an impact on survival outcomes^15,22^. Patients with ΔSUV > 60% after neoadjuvant CCRT showed better OS and disease-free survival^15^. However, a systematic review of the predictive value of PET-CT for tumor response, based on 20 reports, showed variable ranges in sensitivity (33–100%), specificity (30–100%), and differences in PPV and NPV^14^. Hence, prediction of treatment response based on PET-CT parameters alone may be risky as the level of accuracy reported in the literature varies considerably.

To improve the predictive value of treatment response, we herein combined ΔNLR and ΔSUV ratio. Inflammation stimulates angiogenesis, influences immune surveillance on tumor cells, and affects treatment response^10^. As a marker for systemic inflammation, low NLR reflects a low neutrophil count and a high lymphocyte count. Neutrophils are associated with the production of various inflammatory mediators, which are related to systemic inflammation. Lymphocytes are crucial in cell-mediated immunity to initiate tumor cell death. Low NLR represents a low level of systemic inflammation and better cell-mediated immunity toward tumor cells and consequently better response to treatment.

Generally, the intensity of tumor proliferation is correlated with its glucose metabolic activity. Tumor proliferation affects the clinical behavior, which determines prognosis and treatment response. In clinical practice, PET/CT with a radiolabeled glucose analog can detect glucose metabolic activity, which helps to predict prognosis in several malignancies^23^. Overexpression of glucose transporter 1 (GLUT-1) in SCC was associated with a higher FDG-uptake and a negative survival impact^24^. In esophageal SCC, FDG-PET/CT was a useful tool for predicting survival and an indicator of residual tumor burden before surgery^15^. Greater drops in FDG-uptake, measured as SUV after treatment, reflect a decline in tumor metabolism of glucose, or a better response to treatment. Both NLR and SUV are therefore predictors of anticancer treatment response.

Esophagectomy is a major surgical operation associated with morbidities such as leakage (10%), mortality (3 to 5%), and impairment of life quality^9^. Elderly patients with multiple comorbidities may have higher risks of post-esophagectomy complications^25^. A more accurate preoperative assessment of treatment response, such as the ΔNLR and ΔSUV ratio, would allow surgeons to make an individualized treatment plan for their patients. Those who are at high risk for postoperative complications, but have a high probability of achieving a pCR, could potentially be spared the morbidity and mortality of surgery. The organ-preserving approach with active surveillance is an alternative treatment for these patients^26–28^. Van der Wilk et al. reported that patients with esophageal cancer who achieved a clinical complete response after neoCRT and underwent active surveillance had a survival outcome comparable to immediate surgery after neoCRT. The 3-year OS was 77% vs. 55% (p = 0.104) and the 3-year PFS was 60% v. 54% (p = 0.871) in the active surveillance and immediate surgery groups, respectively^27^. Taketa et al. described similar findings. The median OS was not statistically different between the patients who declined surgery (DS) and those who received surgery (trimodality therapy, TMT) after neoCRT (57.9 months v. 50.8 months, p = 0.28). There was a favorable trend toward TMT in relapse-free survival (RFS), but the difference in 3-year RFS was not statistically significant (23.8% v. 45.2%, p = 0.45)^28^.

This study had some limitations. The investigation was retrospective in nature, was conducted in a single institution, and the sample size was relatively small in 9 years. One advantage of this study was the highly homogenous treatment protocol that was applied in all reviewed patients. Another advantage was the high compliance of patients who received neoCRT. All of our patients completed radiotherapy and two cycles of concurrent chemotherapy.

## Conclusion

Change of both NLR and SUV ratio after neoCRT were good predictors of pCR. This finding may help decision-making with respect to the organ-preserving approach in elderly patients with comorbidities.

## Supplementary Information

Supplementary Information.

## Data Availability

The datasets generated during and/or analyzed during the current study are available from the corresponding author on reasonable request.
