# Supplementary material for: Good prediction of treatment responses to neoadjuvant chemoradiotherapy for esophageal cancer based on preoperative inflammatory status and tumor glucose metabolism
Source: Sci Rep. 2021 Jun 2;11:11626. doi: 10.1038/s41598-021-90753-y (PMC8172631; doi:10.1038/s41598-021-90753-y)

Good Prediction of Treatment Responses on Esophageal Cancer after Neoadjuvant Chemoradiotherapy Based on Preoperative Inflammatory Status and Tumor Glucose Metabolism

Chuan Li, M.D. [1], Hui-Ling Yeh, M.D.[1], Jing-Wei Lin, M.D. [1], Cheng-Yen Chuang, M.D., Ph.D[3], Chien-Chih Chen, M.D., Ph. D.[1,2,*]

Supplement 1, ROC curve

(a)ΔSUV

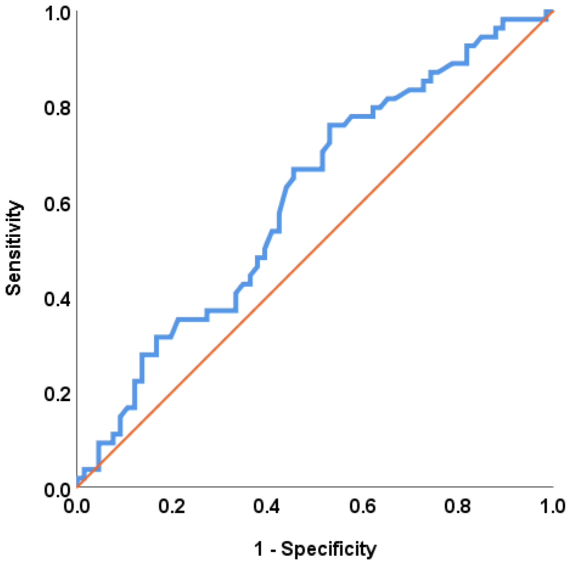

(b) ΔSUV ratio

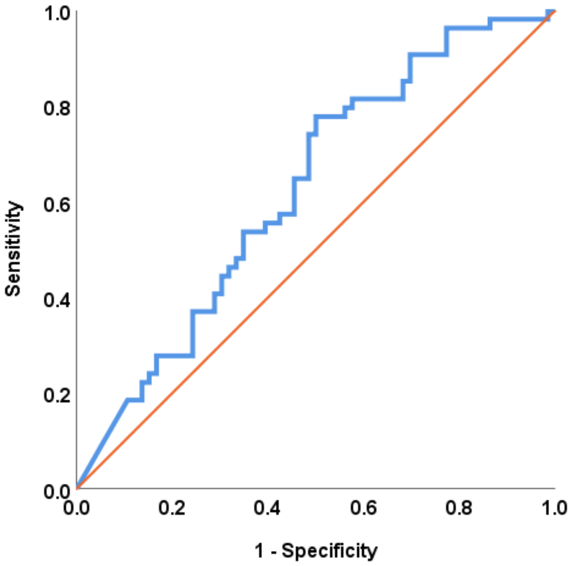

(c) PreOP NLR

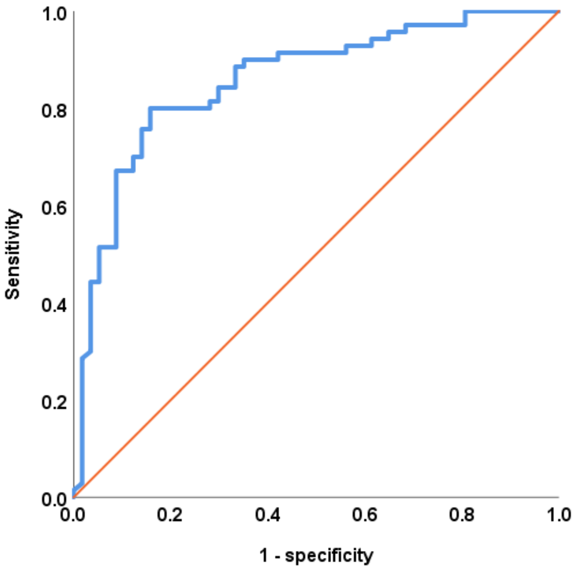

(d) ΔNLR

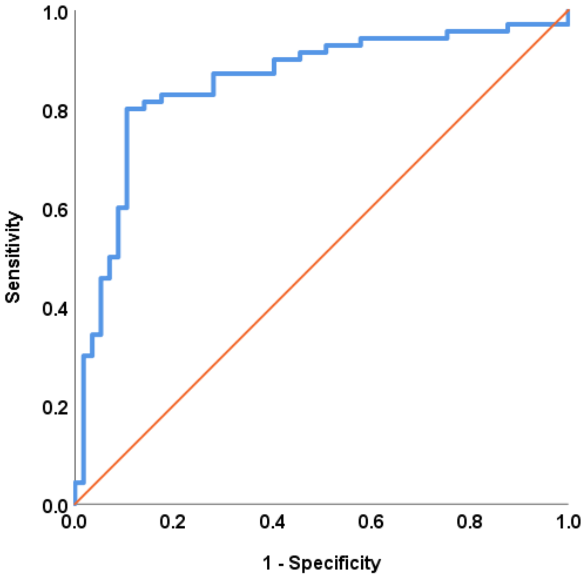

Supplement: Supplementary file 1 — Supplementary Information. [file 41598_2021_90753_MOESM1_ESM.pdf]
